# IgG Anti-High Density Lipoprotein Antibodies Are Elevated in Abdominal Aortic Aneurysm and Associated with Lipid Profile and Clinical Features

**DOI:** 10.3390/jcm9010067

**Published:** 2019-12-26

**Authors:** Javier Rodríguez-Carrio, Jes S. Lindholt, Marina Canyelles, Diego Martínez-López, Mireia Tondo, Luis M. Blanco-Colio, Jean-Baptiste Michel, Joan Carles Escolà-Gil, Ana Suárez, José Luis Martín-Ventura

**Affiliations:** 1 Area of Immunology, Department of Functional Biology, University of Oviedo and Instituto de Investigación Sanitaria del Principado de Asturias (ISPA), 33006 Oviedo, Spain; rodriguezcjavier@uniovi.es; 2 Centre of Individualized Medicine in Arterial Disease (CIMA), Department of Cardiology, Odense University Hospital, 5000 Odense, Denmark; 3 Institut d’Investigacions Biomèdiques (IIB) Sant Pau and CIBERDEM, 08041 Barcelona, Spain; 4 IIS-Fundación Jiménez Díaz and CIBERCV, 28040 Madrid, Spain; 5 INSERM U1148, X. Bichat Hospital, 75018 Paris, France

**Keywords:** abdominal aortic aneurysm, autoantibodies, autoimmunity, HDLc

## Abstract

High-density lipoproteins cholesterol (HDLc) levels are decreased in abdominal aortic aneurysm (AAA), which is hallmarked by autoimmunity and lipid aortic deposits. To investigate whether IgG anti-HDL antibodies were present in AAA and their potential association with clinical features, IgG anti-HDL and total IgG along with HDLc plasma levels were measured in 488 AAA patients and 184 controls from the Viborg Vascular (VIVA) study, and in tissue-conditioned media from AAA intraluminal thrombus and media layer samples compared to control aortas. Higher IgG anti-HDL levels were found in AAA compared to controls, even after correcting for total IgG, and after adjusting for potential confounders. IgG anti-HDL levels were correlated with aortic diameter in univariate and adjusted multivariate analyses. IgG anti-HDL antibodies were negatively associated with HDLc levels before and after correcting for potential confounders. Increased anti-HDL antibodies were identified in tissue-conditioned media from AAA samples compared to healthy aortas, with higher levels being observed in the media layer. In conclusion, increased IgG anti-HDL levels (both in plasma and in tissue) are linked to AAA, associated with aortic diameter and HDLc levels. These data suggest a potential immune response against HDL in AAA and support an emerging role of anti-HDL antibodies in AAA.

## 1. Introduction

Abdominal Aortic Aneurysm (AAA) is hallmarked by a permanent, focal dilation of the abdominal aorta [1]. Despite being mainly asymptomatic, AAA rupture is fatal in most of the cases [2]. Therefore, a better understanding of its pathogenic mechanisms could lead to the identification of novel biomarkers to improve patient management and stratification.

Several risk factors have been related to AAA, such as hypertension, smoking, or obesity. Recent studies suggest that blood lipids may play a role in AAA aetiology [3,4,5,6,7]. Decreased levels of High-Density Lipoproteins cholesterol (HDLc) have been associated with AAA development and progression [8,9,10]. Moreover, raising HDLc inhibits AAA formation and progression in experimental models [11,12]. HDLc levels are affected by various lifestyle-related factors including smoking, alcohol drinking, regular exercise, diet, and body weight [13]. Furthermore, HDLc levels tend to fall during acute or chronic inflammation [14]. The observed reduction in HDLc is thought to be due, at least in part, to the upregulation of proinflammatory cytokines by hepatocytes, resulting in reduced expression and secretion of ApoAI, the major component of HDLs [15]. Additionally, oxidation dissociates ApoAI from its lipid cargo [16], promoting its renal clearance [17], thus accounting for the reduction in HDLc levels. However, the exact mechanisms leading to decreased HDLc levels in AAA remain unknown, hence warranting the involvement of emergent players.

Immune mechanisms are involved in AAA [18,19]. AAA patients are characterized by features of autoimmunity and lipid deposits in the wall of diseased aorta [20,21]. Emerging evidence from autoimmune diseases has revealed that the presence of autoantibodies directed against HDLc (IgG anti-HDL) may explain, at least in part, an impaired lipid profile in these conditions and they were associated with a number of disease outcomes [22,23,24,25]. Interestingly, similar results have been found in other non-autoimmune CVD conditions [26,27], hence suggesting that anti-HDL antibodies can be a common, shared mechanism across different conditions. However, whether these antibodies could be found in AAA is still unknown. 

Therefore, in the present study, we aimed (i) to evaluate whether IgG anti-HDLc antibodies are present in AAA patients and (ii) to analyse their potential association with HDLc levels and clinical features.

## 2. Materials and Methods

### 2.1. Patients

This observational study was conducted in the framework of a population-based image-screening trial for AAA in Danish men aged 65–74 years (VIVA, ClinicalTrials.gov NCT00662480) [28]. The study protocol design has been published in detail elsewhere [29].

Briefly, 50,156 male individuals were randomized to either receive an invitation for vascular screening or being a control subject between October 2008 and October 2010. A total of 18,749 individuals out of the 25,078 invited in the vascular screening arm agreed to participate [28]. The study protocol was performed in compliance with the Declaration of Helsinki and approved by the Institutional Review Board from the Mid Region of Denmark (M20080028). All participants gave written informed consent prior enrolment.

At study entry, an ankle-brachial index (ABI) measurement, an ultrasound scan of the aorta as well as a questionnaire regarding lifestyle parameters, medical and smoking status was performed by trained project-nurses in 3 mobile units at local hospitals in the mid region of Denmark.

The visualization of the aorta was performed by ultrasound, by placing the 4 MHz-transducer longitudinally just above and a little to the left of the navel. In cases of dilation, the maximal perpendicular anterio-posterior (AP) diameter was measured. If no dilation is observed, the AP diameter was measured two centimeters above the bifurcation. The ankle systolic blood pressure was also measured, and maximal AP diameter of the infrarenal aorta was measured at the peak of systole from the inner to the inner edge of aorta. The ABI was calculated as the mean of the two recorded ankle arterial blood pressures divided by the brachial systolic blood pressure. The presence of peripheral arterial disease (PAD) was defined by ABI <0.90 or >1.4.

Blood samples were obtained by venipuncture. Plasma samples were immediately processed and stored at −80 °C until laboratory analyses.

### 2.2. Laboratory Analyses

Plasma ApoAI was determined by an immunoturbidimetric assay, using a commercial kit adapted to a COBAS 501c autoanalyzer (Roche Diagnostics, Rotkreuz, Switzerland). HDLc levels were measured in plasma obtained after precipitation of ApoB-containing lipoprotein particles with phosphotungstic acid and magnesium ions (Roche Diagnostics, Rotkreuz, Switzerland).

### 2.3. Tissue-Conditioned Media

Tissue-conditioned media was obtained as described [30]. In brief, tissue samples were collected during surgical repair from AAA patients and dissected into intraluminal thrombus and wall (media layer). Healthy abdominal aortas were sampled from brain-deceased organ donors during organ removal for therapeutic transplantation (kidney or liver transplantation). The aortic tissue was washed and preserved in ringer lactate solution at 4 °C until use, with a time frame between sampling and freezing always less than 6 h. Then, tissue sections were cut into small pieces (5 mm^2^) and incubated in RPMI 1640 medium containing antibiotics and an antimycotic (Gibco) for 24 h at 37 °C (6 mL/g of wet tissue). The conditioned media (supernatants containing proteins released by the tissue samples) were obtained after centrifugation (3000 g for 10 min at 20 °C) and kept at –80° until further processed. The adequacy of this protocol to ensure optimal tissue preservation was previously evaluated and reported by our group [31], and used thereafter in the literature. Ethical committee advice and patient informed consent were obtained (RESAA and AMETHYST studies, CPP Paris-Cochin n° 2095, 1930 and 1931, INSERM Institutional Review Board, IRB0000388). Healthy abdominal aortas were obtained with the authorization of the French Biomedicine Agency (PFS 09-007, BBMRI network, BB-0033-00029).

### 2.4. Measurement of IgG Antibodies against HDL

IgG anti-HDL antibodies were measured in all plasma samples by means of an in-house immunoassay as previously reported [23]. In brief, ELISA plates (Maxisorp, Nunc, Germany) were coated with either 20 mg/mL human HDL-cholesterol (unfractioned HDL isolated from human plasma, Sigma, Germany) in 70% ethanol (test half) or ethanol alone (control half) overnight at 4 ºC. Plates were then blocked with PBS + 1% BSA (Sigma) for 1 h at room temperature, washed with PBS, and 1:50-diluted plasma samples and standard curves from pooled sera (diluted 1:16 to 1:512) were incubated in both plate halves for 2 h at room temperature. Next, plates were washed twice with TBS and alkaline phosphatase-conjugated anti-human IgG (1:1000) (Immunostep, Spain) was added for 1 h. Finally, p-nitrophenylphosphate (Sigma) in diethanolamine buffer (pH 9.8) was added and absorbance at 405 nm was recorded. IgG anti-HDL Arbitrary Units (AU) were calculated for each sample according to the standard curves. Intra- and inter-assay reproducibility for our assay was 10% and 13%, respectively, using lower limit of quantification, low, medium and high quality control samples (*n* = 4/group) within a single run and between different runs under identical experimental conditions. The same protocol was used for tissue-conditioned media, which were assayed undiluted and the raw absorbances were analyzed.

Total IgG plasma levels were quantified by conventional ELISA techniques and AU values obtained from the anti-HDL ELISA were corrected using total IgG levels (anti-HDL/IgG). The positivity to anti-HDL antibodies was evaluated using quartiles: those with undetectable IgG anti-HDL/IgG levels or below first quartile were considered without anti-HDL antibodies, whereas those with levels above first quartile were considered to have anti-HDL antibodies. This categorization was intended to evaluate the anti-HDL burden in our study individuals but all the analyses were performed with the whole group.

### 2.5. Statistical Analyses

Normal distribution of data was assessed using graphical methods and the Shapiro–Wilk test. Residuals were assessed in histograms and p–p plots. Levels of IgG anti-HDL were log-transformed to gain an acceptable normal distribution before being entered in parametric and multivariate tests. Continuous variables were summarized as median (interquartile range) or mean ± standard deviation (SD) depending on the distribution of the data. Categorical variables were expressed as *n* (%). Differences between AAA and controls in main clinical characteristics and laboratory analyses were assessed by Student’s *t* test. Independent associations between AAA prevalence and IgG anti-HDL antibodies were assessed by logistic regression analyses with adjustments for active smoking, hypertension, use of statins, use of low dose aspirin, BMI, systolic blood pressure, and PAD at screening. Selection of confounders was performed by identifying those associated with the dependent variable (AAA presence, aortic size, HDLc levels) with a *p*-value below 0.1 in univariate analyses [32,33].

Additionally, the Pearson’s correlation coefficient was used to study the association between IgG anti-HDL antibodies and maximal aortic diameter and ABI. The association between IgG anti-HDL levels and aortic diameter was further studied by multivariate regression analyses adjusted for active smoking, hypertension, atherosclerosis occurrence, BMI, diastolic blood pressure, use of statins, use of low dose aspirin and PAD at screening.

Finally, the association between IgG anti-HDL antibodies and HDLc was tested in linear regression analysis with and without adjustment for active smoking, hypertension, use of statins, use of low dose aspirin, BMI, diastolic blood pressure, and PAD at screening.

## 3. Results

### 3.1. Plasma Levels of IgG Anti-HDL Antibodies Are Increased in AAA Patients

**Figure 1:**
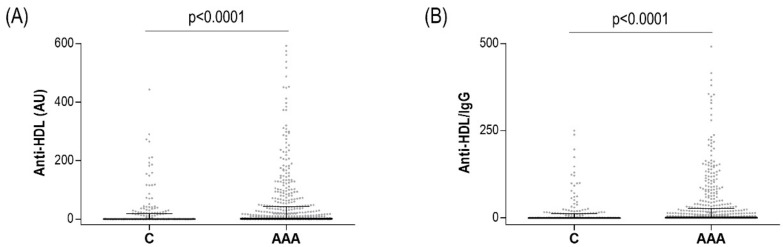
IgG anti-HDL antibodies in AAA. IgG anti-HDL plasma levels measured as AU (**A**) or normalized after total IgG correction (**B**) in AAA patients (*n* = 488) and healthy controls (*n* = 184). Bars indicate 25th, median and 75th percentiles. Differences were assessed by Mann–Whitney U tests.

Characteristics of the studied population are summarized in Table 1. Plasma levels of IgG anti-HDL antibodies were significantly increased in AAA patients (*n* = 488) compared to age-matched AAA-free controls (*n* = 184) (Figure 1A). These differences remained even after correcting for total IgG levels (anti-HDL/IgG) (Figure 1B). No differences in total IgG serum levels were observed between AAA and controls (1.80 ± 1.06 vs. 1.69 ± 0.80 AU, *p* = 0.906). Then, 197 (40.3%) AAA patients were classified as anti-HDL-positive, compared to 58 controls (31.5%, *p* = 0.035), hence confirming a higher anti-HDL burden in AAA patients compared to controls.

The levels of anti-HDL antibodies were not associated with age (*r* = 0.019, *p* = 0.823), body mass index (BMI) (*r* = 0.040, *p* = 0.645), hypertension (*p* = 0.343), smoking (*p* = 0.563) and use of medications (all *p* >0.050). Moreover, the levels of anti-HDL antibodies did not differ between patients with spontaneous AAA (*n* = 451) and those with familiar history (*n* = 37; *p* = 0.815). In AAA patients, IgG anti-HDL antibodies were negatively associated with HDLc levels before (*r* = −0.093, *p* = 0.009) (Supplementary Figure S1) and after adjustment for traditional CV risk factors as confounders (Table 2). No significant association was observed between anti-HDL/IgG and ApoAI (*r* = −0.042, *p* = 0.273).

The presence of IgG anti-HDL was independently associated with AAA after correcting for traditional CV risk factors and potential confounders (smoking, hypertension, systolic BP, BMI, ABI, PAD, use of statin, use of low dose aspirin, and use of beta-blockers) (Table 3). Similarly, adjusting for hsCRP did not change the independent association between the presence of IgG anti-HDL levels and AAA (OR [95% CI]: 2.456 [1.0558–3.872], *p* <0.001).

Furthermore, IgG anti-HDL levels were correlated with aortic diameter in AAA patients in univariate (*r* = 0.122, *p* = 0.001) and multivariate analyses adjusted for potential confounders (smoking, hypertension, systolic BP, BMI, PAD, use of statins, use of low-dose aspirin and use of beta-blockers) (Table 4). This association remained after including hsCRP in the model (B [95% CI]: 1.344 [0.047–2.641], *p* = 0.042).

Variables were summarized as mean ± SD, median (25th percentile–75th percentile), or *n* (%). Differences between groups were assessed by Chi Square for categorical variables and unpaired *t*- test for numerical ones. ^‡^ hsCRP levels were available in 460 AAA patients and 169 controls.

### 3.2. IgG Anti-HDL Antibodies Can Be Detected in AAA Tissue-Conditioned Media

Next, to evaluate whether IgG anti-HDL could be detected in situ, we used tissue-conditioned media samples obtained from AAA (Intraluminal Thrombus, T: *n* = 10 and media layer, M: *n* = 10) and also healthy aorta (H: *n* = 10) (Supplementary Table S1).

**Figure 2:**
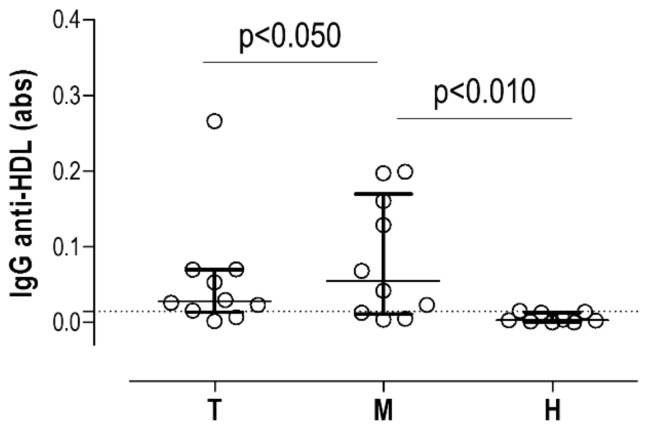
IgG anti-HDL antibodies in tissue-conditioned media. IgG anti-HDL plasma levels (measured as absorbance) in tissue-conditioned media samples obtained from thrombus (T, *n* = 10) and media (M, *n* = 10) samples from AAA subjects and healthy (H, *n* = 10) aortas. Bars indicate medians, whereas whiskers represent 25th and 75th percentiles. Horizontal dotted line represents 90th percentile from healthy samples. Differences were assessed by Kruskal–Wallis tests (*p* = 0.0030) and p-values from Dunn–Bonferroni multiple comparisons tests are indicated in the figure.

Interestingly, IgG anti-HDL were clearly detected in tissue-conditioned media from AAA (M: 8/10 samples over 90th percentile value in healthy samples, and T: 8/10) compared to those from healthy aortas (H: 1/10) (Figure 2). Moreover, higher levels of IgG anti-HDL antibodies were found in tissue-conditioned media from AAA media compared to the thrombus (Figure 2).

## 4. Discussion

Although epidemiological evidence supports a negative association of HDLc with AAA, and a number of studies point to an altered lipid profile in AAA patients, the connection between CVD and HDLc in AAA is more complex than initially conceived. The results herein presented suggest that anti-HDL antibodies may be involved, at least in part, in this scenario. To the best of our knowledge, this is the first study that characterizes the presence of anti-HDL antibodies in AAA (both in plasma and in tissue), their negative association with HDLc levels as well as with clinical features of AAA.

Altered lipid profiles in AAA patients have been widely reported [8,9,10,34]. Additionally, traditional CV risk factors seem to perform sub-optimally in capturing the real risk, hence suggesting the involvement of additional, non-traditional, factors. Then, the role of immune-inflammatory responses in AAA is well accepted [35,36], but the critical immune mediators are poorly characterized. There is relatively solid knowledge on the cellular components of the immune responses in AAA [37,38,39]. Interestingly, a shift towards a Th2 response has been reported [40], although other reports have found a prominent Th1 profile [41]. Furthermore, the role of the humoral response remains unclear. In this respect, the relevance of autoantibodies for CVD has been previously appreciated [42,43], but there is scarce data regarding AAA [44]. Our findings add a new layer of evidence to this setting and expand the current knowledge on autoantibodies, namely anti-HDL antibodies, in AAA. The presence of anti-HDL antibodies was independent of traditional risk factors, hence strengthening their role as additional, non-traditional risk factors. Moreover, the presence of anti-HDL antibodies was not attributed to a generalized IgG production, but to a specific production of these antibodies. Moreover, the detection of anti-HDL antibodies at the local level in tissue-conditioned media from AAA patients reinforces its potential role as pathogenic mediators in this setting.

A main finding of our study is the negative association of anti-HDL antibodies with HDLc levels in AAA patients. Interestingly, the same does not hold true for ApoAI levels, highlighting the immunogenic relevance of HDL components other than ApoAI in the humoral response against native/unmodified HDL. Similar results have been reported elsewhere in autoimmune [25,45] and non-autoimmune conditions [26], thus suggesting that anti-HDL measurement captures a much more complete immunological picture compared to individual specificities, which can in turn have an additional clinical value. Moreover, it is important to note that changes in HDL protein composition have been described in AAA [46,47], and other conditions [48]. Consequently, a more global approach may be more appropriate than a single antigen-restricted one, thereby reinforcing the adequacy of an anti-HDL measurement.

Another interesting finding from our study was the association between anti-HDL antibodies and clinical features, such as aortic diameter, a surrogate marker of AAA progression. Far from being innocent bystanders, autoantibodies are complex molecules with pleiotropic activities, including activation of immune cells bearing Fc receptors, leukocyte activation following immune complex deposition, complement fixation, and promoting tissue remodeling [42], which can explain the effects at the systemic level. As previously commented, anti-HDL antibodies have been connected to inflammatory pathways [22,24,27,49], which play a role as modulators of AAA progression [50]. Taken together, it is tempting to hypothesize that anti-HDL antibodies may be one of the missing links between autoimmunity and AAA pathogenesis. The fact that these antibodies could be detected in the media layer of the AAA tissues, where a major cholesterol deposition is observed [18], sheds new light on the contribution of this local area as a potential trigger for anti-HDL production in AAA.

Although this study paved the way for the analysis of HDL-targeted autoimmune phenomena in AAA, since all the patients exhibited AAA at the time of sampling, the possibility that these antibodies emerge as a consequence of the AAA development instead of as a pre-existing mediator cannot be ruled out. However, it has been reported in the literature that these antibodies are present in several risk scenarios [51,52], and even in the general population [53,54], and that they preceded disease outcomes. Ultimately, the clinical validation of the plasma anti-HDL as a biomarker of AAA will come from the association with future AAA expansion and/or rupture studies, a major unmet clinical need [55] and an area of research with major interest from the clinical perspective [56]. Similarly, the detection of these antibodies may provide additional clues to understand the risk stratification and risk target attainment in these patients, which represents a key aspect of the clinical management. Moreover, anti-HDL may also be considered as additional diagnostic/prognostic tools to CV risk factors and/or aortic size in the clinical management of AAA patients. In fact, several studies have demonstrated that these antibodies improve patient classification and risk stratification in several CV risk-related scenarios over traditional algorithms [26,57,58]. Importantly, the detection of antibodies is a relatively simple technique, objective, reproducible and feasible in most laboratory hospitals, which provides additional advantages over more sophisticated and less accessible techniques, such as imaging. However, it is important to note that cost-effectiveness analyses must be taken into consideration to fully elucidate the potential role as a biomarker of these antibodies, in light of the results herein reported.

This study has some limitations that should be remarked. Due to the cross-sectional design, a cause–effect association between anti-HDL and HDLc cannot be confirmed. Moreover, the effect observed was moderate, which requires a validation in an independent cohort and additional studies to evaluate their potential role as a biomarker. Similarly, the potential effect of some treatments on anti-HDL levels cannot be assessed in this study. Finally, it may be interesting to expand the analysis of anti-HDL levels in tissue-conditioned media in order to evaluate whether local anti-HDL antibody production correlates with clinical features as well. The sample size used in this study was sufficient to evaluate the differences in levels but prevented us from assessing associations with clinical features, which were beyond the scope of this study. However, this study has important strengths, because of its sample size, robust epidemiological methodology, population-based design and an adequate analysis of potential confounders.

In conclusion, increased IgG anti-HDL serum and tissue levels are observed in AAA patients. These antibodies negatively correlated with HDLc and positively correlated with aortic diameter in a large, population-based study. Collectively, our data suggest a potential humoral immune response against HDL particles in AAA. From a basic perspective, the investigation of the specific epitopes targeted by these antibodies must be placed in the future research agenda. From a clinical point of view, further research to elucidate the exact role of anti-HDL antibodies as biomarkers for patient stratification and/or clinical management, as well as their contribution the mechanisms of AAA, is warranted.

## Figures and Tables

**Table 1:** Demographical and clinical parameters of the study subjects.

	AAA (*n* = 488)	Controls (*n* = 184)	*p*-Value
Age	70.0 ± 2.8	69.6 ± 2.9	0.411
Sex, *n* (%) male	488 (100)	184 (100)	-
BMI, kg/cm^2^	27.4 ± 3.6	26.3 ± 3.3	0.021
ABI	0.9 ± 0.2	1.1 ± 0.1	0.004
Aortic size, mm	40.9 ± 11.8	18.2 ± 2.8	<0.001
PAD, *n* (%)	122 (25.2)	5 (2.7%)	<0.001
hsCRP, mg/L ^‡^	3.00 (1.60–6.30)	1.60 (0.90–3.75)	<0.001
*Lipid profiles, mmol/L*			
Total-cholesterol	4.88 ± 0.91	4.84 ± 1.14	0.592
HDL-cholesterol	1.17 ± 0.41	1.33 ± 0.45	<0.001
ApoAI	1.58 ± 0.29	1.72 ± 0.32	<0.001
*CV risk factors*			
Current smoking, *n* (%)	207 (42.4)	34 (18.5)	<0.001
Hypertension, *n* (%)	265 (54.4)	82 (45.3)	0.036
Systolic blood pressure, mm Hg	155.4 ± 21.5	147.4 ± 19.2	0.021
Diabetes, *n* (%)	57 (11.7)	28 (15.3)	0.209
*Treatments, n(%)*			
Use of statins	250 (51.9)	67 (36.4)	<0.001
Use of low-dose aspirin	228 (47.2)	46 (25.0)	<0.001
Use of b-blockers	139 (29.1)	40 (21.6)	0.051

**Table 2:** Multivariate regression analysis to evaluate the role of anti-HDL/IgG as independent predictors of HDLc levels.

	B	95% CI	*p*-Value
IgG anti-HDL/IgG	−0.054	−0.094–−0.013	0.009
Current smoking, yes	−0.099	−0.170–−0.027	0.007
Hypertension, yes	0.029	−0.040–0.099	0.665
Systolic BP, per unit	0.001	−0.002–0.004	0.629
BMI, per unit	−0.026	−0.035–−0.016	<0.001
PAD, yes	−0.052	−0.139–0.035	0.244
Use of statins, yes	0.081	0.002–0.160	0.243
Use of low-dose aspirin, yes	−0.039	−0.117–0.039	0.326

**Table 3:** Multivariate logistic regression analysis to evaluate the role of anti-HDL/IgG as independent predictors of AAA. The presence of anti-HDL was entered as a dichotomous variable using the objective cut-off explained in the Methods’ section to evaluate the strength of the association between the presence of anti-HDL and AAA.

	B	S.E.	OR	95% CI	*p*-Value
Anti-HDL/IgG, yes	0.915	0.215	2.496	1.637–3.807	<0.001
Current smoking, yes	1.479	0.522	4.387	2.661–7.234	<0.001
Hypertension, yes	0.187	0.259	1.206	0.726–2.003	0.469
Systolic BP, per unit	0.068	0.011	1.070	1.048–1.093	<0.001
BMI, per 1 kg/m^2^	0.097	0.033	1.102	1.032–1.176	0.004
ABI, per unit	−7.401	1.374	0.001	0.001–0.009	<0.001
PAD, yes	2.568	0.255	13.044	4.530–37.562	<0.001
Use of statins, yes	0.265	0.252	1.303	0.796–2.135	0.292
Use of low-dose aspirin, yes	0.977	0.267	2.656	1.575–4.481	<0.001
Use of b-blockers, yes	0.002	0.268	1.002	0.592–1.695	0.994

**Table 4:** Multivariate linear regression analysis to evaluate the role of anti-HDL/IgG as independent predictors of aortic diameter in AAA.

	B	95% CI	*p*-Value
Anti-HDL/IgG	1.480	0.233–2.727	0.020
Current smoking, yes	3.699	1.504–5.894	<0.001
Hypertension, yes	1.962	−0.467–4.390	0.113
Systolic BP, per unit	0.293	0.203–0.381	<0.001
BMI, per 1 kg/m^2^	0.455	0.161–0.749	0.002
PAD, yes	2.338	−0.366–5.042	0.090
Use of statins, yes	1.934	−0.520–4.387	0.122
Use of low-dose aspirin, yes	4.451	1.959–6.944	<0.001
Use of b-blockers, yes	0.443	−2.060–2.947	0.728

## Supplementary Materials

The following are available online at https://www.mdpi.com/2077-0383/9/1/67/s1, Table S1: Demographical parameters of the individuals recruited for the tissue-conditioned media experiments. Figure S1: Association between anti-HDL and HDLc levels.
