# Supplementary material for: IgG Anti-High Density Lipoprotein Antibodies Are Elevated in Abdominal Aortic Aneurysm and Associated with Lipid Profile and Clinical Features
Source: J Clin Med. 2019 Dec 26;9(1):67. doi: 10.3390/jcm9010067 (PMC7019833; doi:10.3390/jcm9010067)
Supplement: Supplementary file 1 [file jcm-09-00067-s001.pdf]

Supplementary Table 1: Demographical parameters of the individuals recruited for the tissue-conditioned media experiments.

	M (n = 10)	T (n = 10)	H (n = 10)	*p*-value
Age	65.0 ± 6.5	62.1 ± 5.8	58.4 ± 8.2	0.117
Sex, n male	10 (100)	10 (100)	7 (77.7)‡	0.092

Variables were summarized as mean ± SD or n(%). ‡ Information available from 9 subjects. Differences between groups were assessed by Chi Square for categorical variables and unpaired t- test for numerical ones.

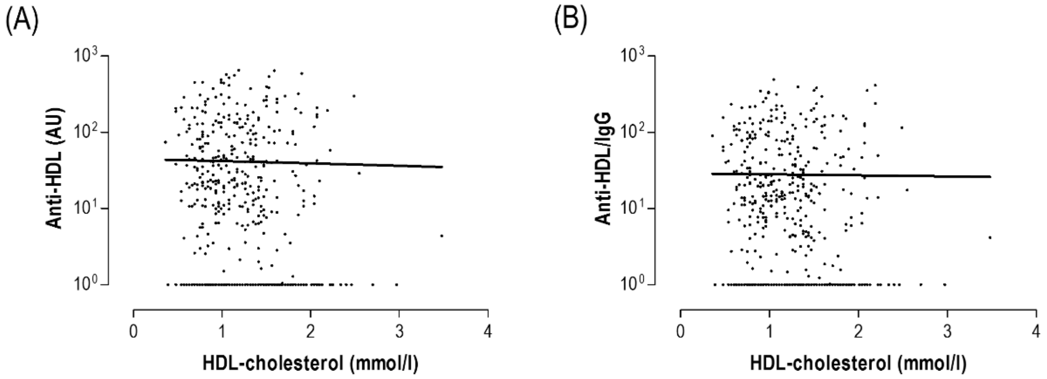

Supplementary Figure 1: Association between anti-HDL levels and circulating HDLc. The univariate association between anti-HDL measured as AU (**A**) or corrected by total IgG (**B**) and circulating HDL levels in AAA patients is shown.
